# Supplementary material for: Bright Tm3+-based downshifting luminescence nanoprobe operating around 1800 nm for NIR-IIb and c bioimaging
Source: Nat Commun. 2023 Feb 25;14:1079. doi: 10.1038/s41467-023-36813-5 (PMC9968279; doi:10.1038/s41467-023-36813-5)
Supplement: Supplementary file 1 — Supplementary Information [file 41467_2023_36813_MOESM1_ESM.docx]

**Supplementary Information**

**Bright Tm^3+^-based downshifting luminescence nanoprobe operating around 1,800 nm for NIR-IIb and c bioimaging**

Yulei Chang^1,^*, Haoren Chen^1^, Xiaoyu Xie^1^, Yong Wan^1^, Qiqing Li^1^, Fengxia Wu^1,2^, Run, Yang^1^, Wang Wang^1^, Xianggui Kong^1^

*Correspondence should be addressed to yuleichang@ciomp.ac.cn

**Materials**

**Reagents**

LiOH·H_2_O, TmCl_3_·6H_2_O, ErCl_3_·6H_2_O, oleic acid (OA, 90%), lithium trifluoroacetate (98%), 1-octadecene (ODE, 90%), (CF_3_COO)_3_Y, and CF_3_COONa were purchased from Sigma Aldrich. Ln_2_O_3_ (Ln: Y, Lu, Gd and Tm > 99.99%), NaOH, NH_4_F, DyCl_3_·6H_2_O and trifluoroacetic acid (99%) were obtained from Aladdin (China). DSPE-PEG was obtained from Ponsure Biological (Shanghai). All chemicals were of analytical grade, and there was no further purification.

**Characterization**

The crystal structure was characterized by using a Bruker D8-advance X-ray diffractometer from 10 to 80° (Cu Kα radiation, λ = 1.5418 Å). The size and morphology of the nanoparticles were performed with a JEOL transmission electron microscope. The scanning electron microscopy (SEM) was determined on a Hitachi S-4800. A SHIMADZU UV-3101PC spectrophotometer measured absorption spectra. The luminescence dynamics were recorded with a 500 MHz Tektronix digital oscilloscope, and the excitation was achieved by a nanosecond pulse train at 800 nm from an optical parametric oscillator. The steady-state luminescence spectra were recorded on an FLS-980 fluorescence spectrometer (Edinburgh Instruments), and the excitation beams were equipped with external 800, 980, 1,208, and 1,530 nm fibre-optic diode laser (CNI Optoelectronics Technology Co. Ltd).


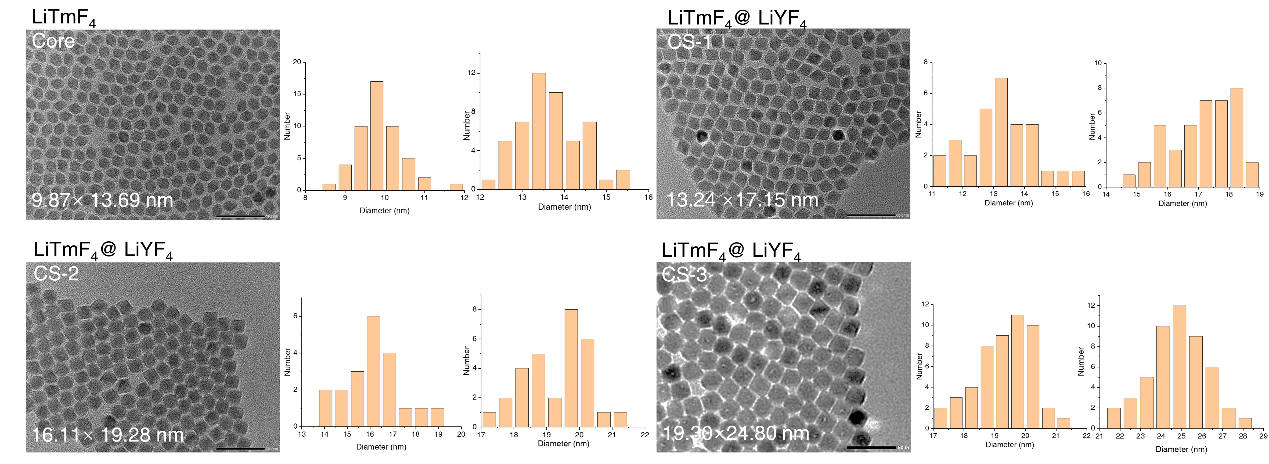


**Supplementary Fig. 1.** TEM images of LiTmF_4_ core and LiTmF_4_@LiYF_4_ core-shell NPs. Scale bar: 50 nm.


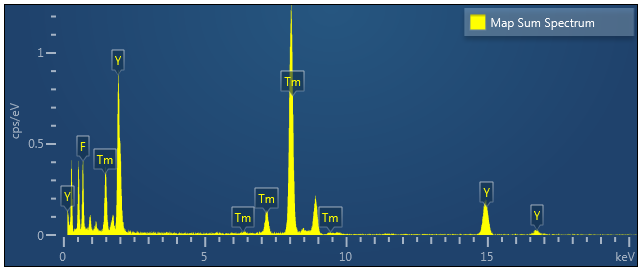


**Supplementary Fig. 2.** EDS pattern of the LiTmF_4_@LiYF_4_ NPs.


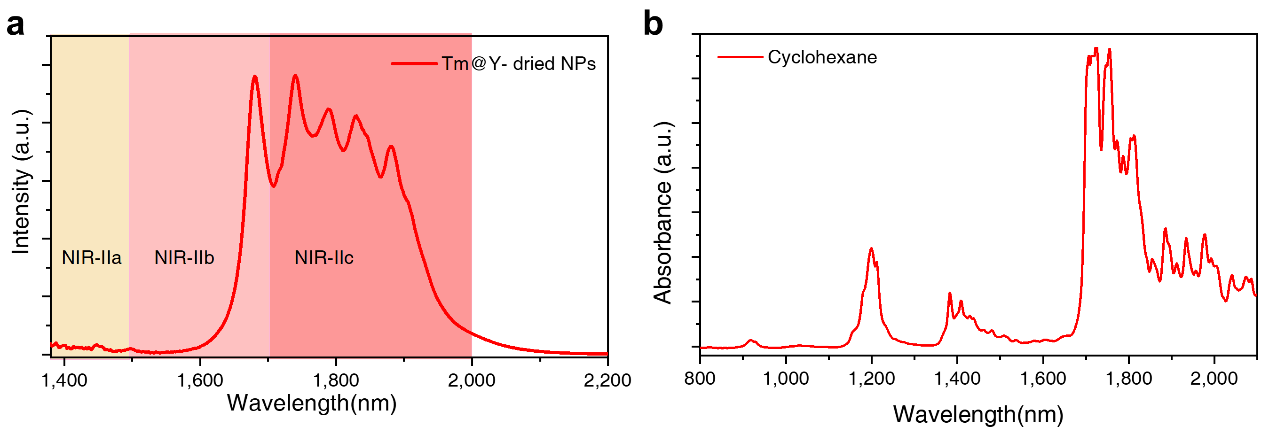


**Supplementary Fig. 3.** **a,** Emission spectra of dried Tm-NPs (dried) under 800 nm light irradiation. **b,** Absorption spectra of cyclohexane.


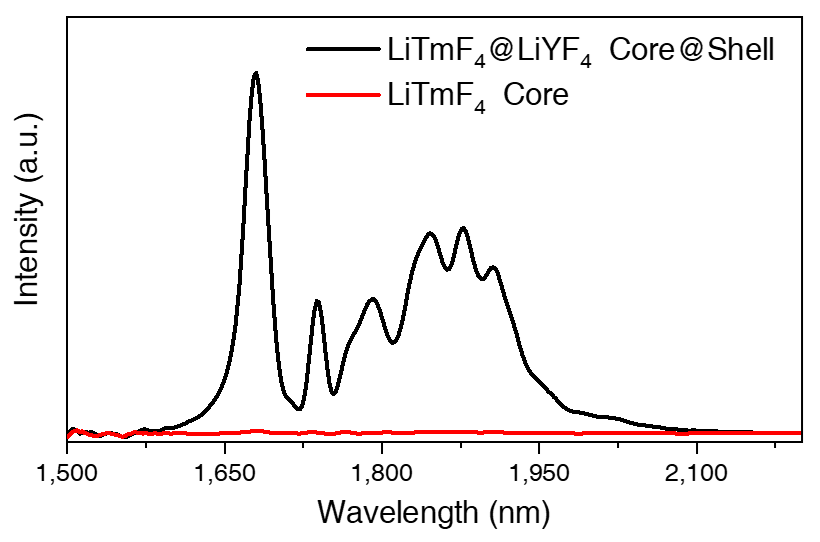


**Supplementary Fig. 4.** Comparison of DSL between LiTmF_4_ core and core-shell Tm-NPs under 800 nm excitation and recorded by the InAs detector.


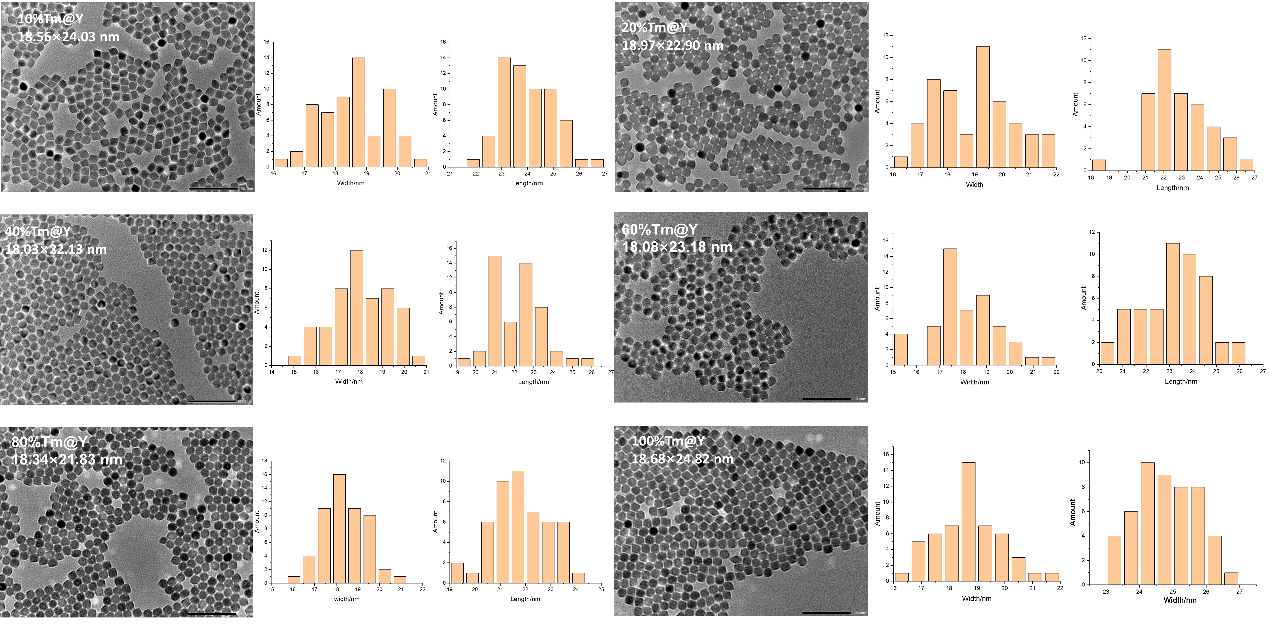


**Supplementary Fig. 5.** TEM images of LiYF_4_: x%Tm@LiYF_4_ core-shell NPs. Scale bars are 100 nm.

## Quantum yield Measurement.

The powder samples of Tm-NPs and Tm(02Er) NPs were excited by an 808 nm laser (CNI, Changchun) at 4 W/cm^2^. An integrating sphere (Labshere, 3.3 inches) was used to spread the multiple light reflections over the entire sphere surface. The spectrometer collected the outcome lights, including excitation light and emission light of samples. The attenuator and inline filter holder (including a 900 nm LP filter) were used during the test. First, calibrate the dual channel spectrometer (QE PRO, 783-1,027 nm, Slit 50 µm and NQ512, 895-2,131 nm, Slit 200 µm) with the calibration light source (HL-3-INT-Cal, ~2,500 nm) to obtain the radiation flux curve. Next, adjust the appropriate integration time for the two spectrometers to enable them to obtain enough effective signals, and then update the background spectrum immediately. Next, spectrometer QE was used to obtain excitation light radiation flux data. Finally, we used spectrometer NQ to obtain the emission light radiation flux data. The software (Ocean QY 2.02) automatically calculated the quantum yield (QY) using the following formula: QY= photons emitted / photons absorbed.


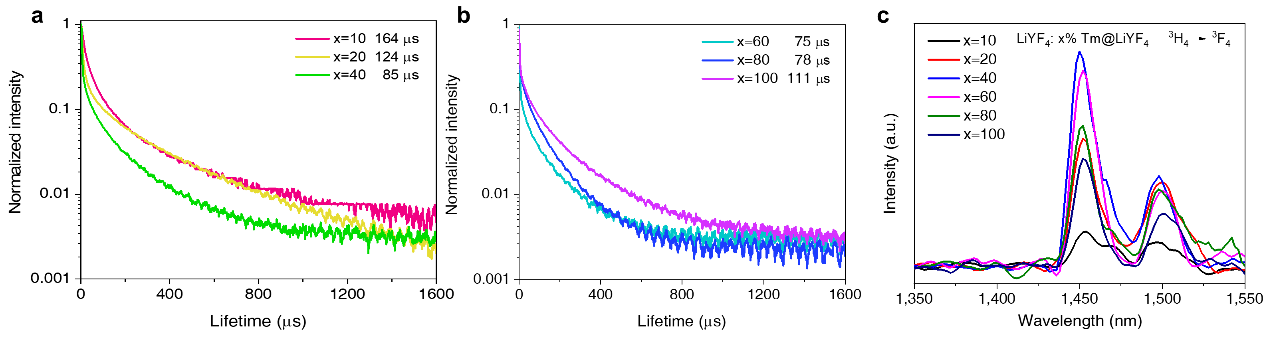


**Supplementary Fig. 6.** Decay time of various concentrations of Tm^3+^. **a,** from 10 to 40%, **b**, from 60% to 100%. **c**, Emission spectra of LiYF_4_:x% Tm@LiYF_4_ at 1,450 nm under 800 nm light irradiation.


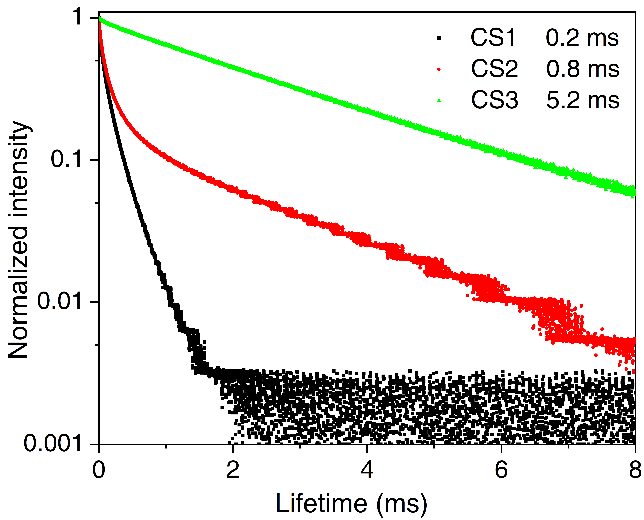


**Supplementary Fig. 7.** Decay time of different shell thicknesses of LiTmF_4_@LiYF_4_.


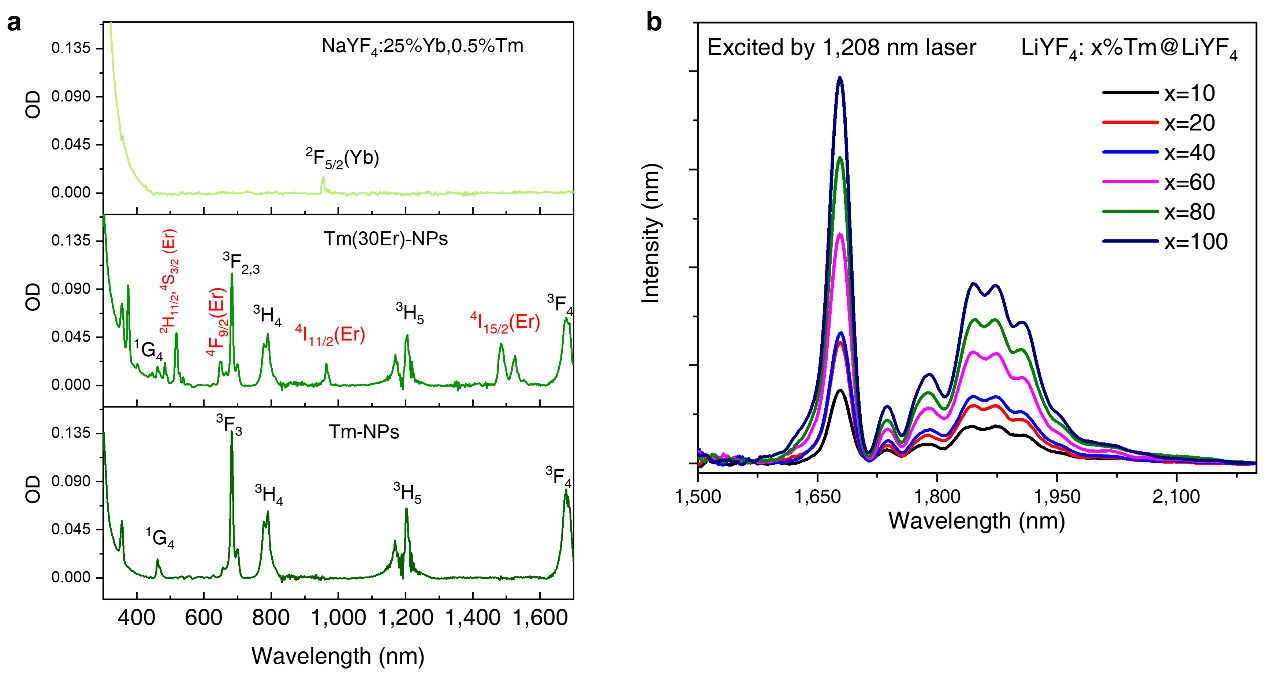


**Supplementary Fig. 8a.** Absorption spectra of NaYF_4_:25%Yb,0.5%Tm, Tm(30Er)-NPs and Tm-NPs in UV-VIS-NIR region. **b**, The emission of LiYF_4_:x% Tm@LiYF_4_ NPs in cyclohexane under 1,208 nm light irradiation (Tm^3+^ doping ratio increases from 10% to 20%, 40%, 60%, 80% and 100%).


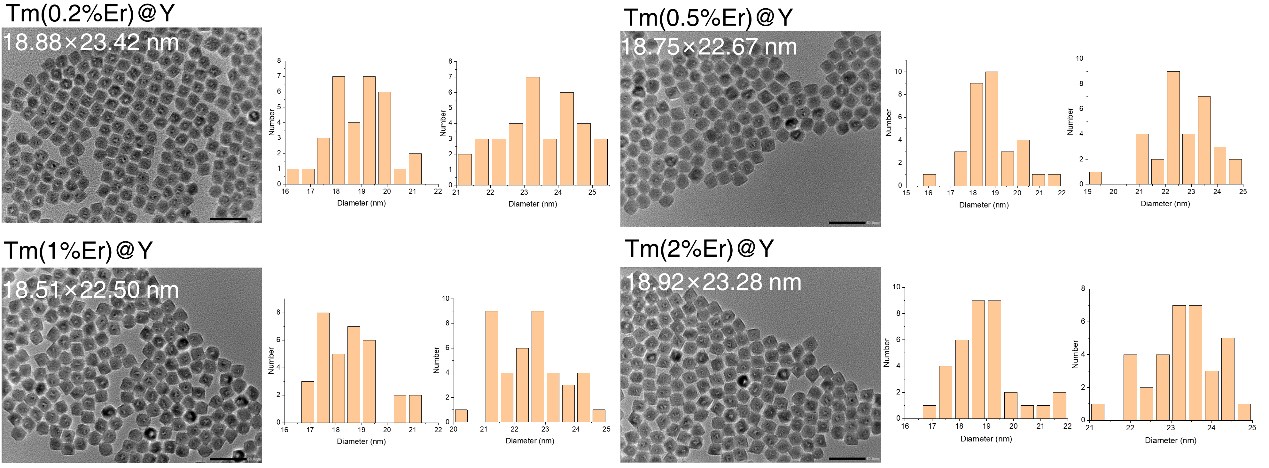


**Supplementary Fig. 9.** TEM images of LiTmF_4_:x%Er@LiYF_4_ core-shell NPs (x=0, 0.2%, 0.5%, 1% and 2 mol%).


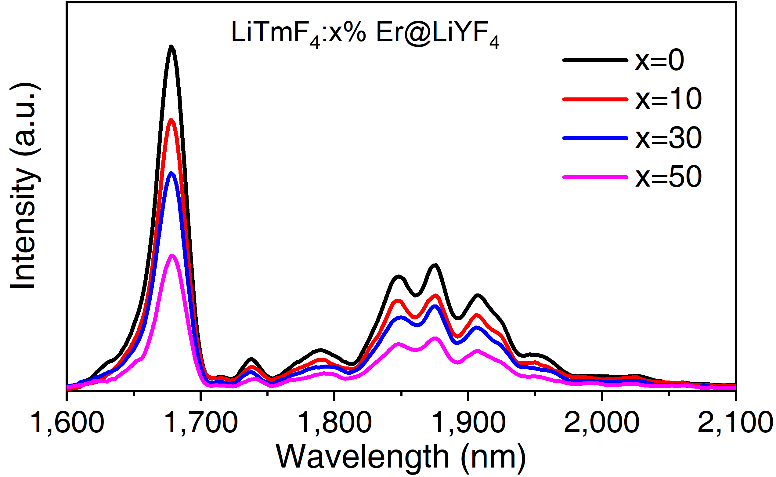


**Supplementary Fig. 10.** Emission spectra of LiTmF_4_:x%Er@LiYF_4_ with Er^3+^ doping level from 10% to 30% and 50 mol% under 800 nm light excitation.


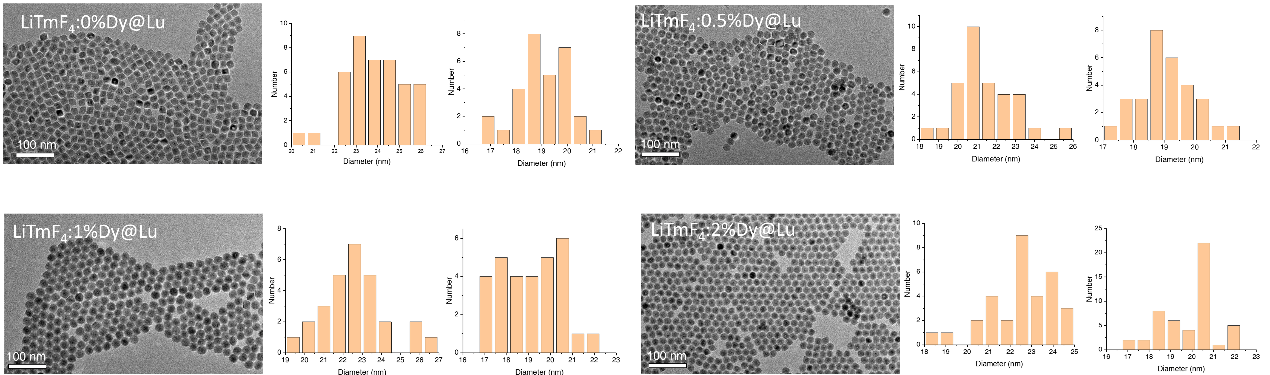


**Supplementary Fig. 11.** TEM images of LiTmF_4_:x%Dy@LiLuF_4_ core-shell NPs. (Dy doping ratio increases from 0, to 0.5%, 1% and 2 mol%), Scale bars are 100 nm.


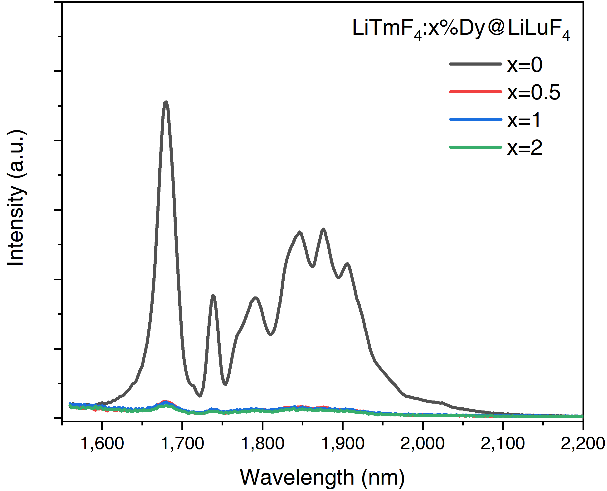


**Supplementary Fig. 12.** Emission spectra of LiTmF_4_:x%Dy@LiLuF_4_ NPs in cyclohexane under 800 nm light irradiation (Dy^3+^ doping ratio increases from 0.5% to 1% and 2 mol%).


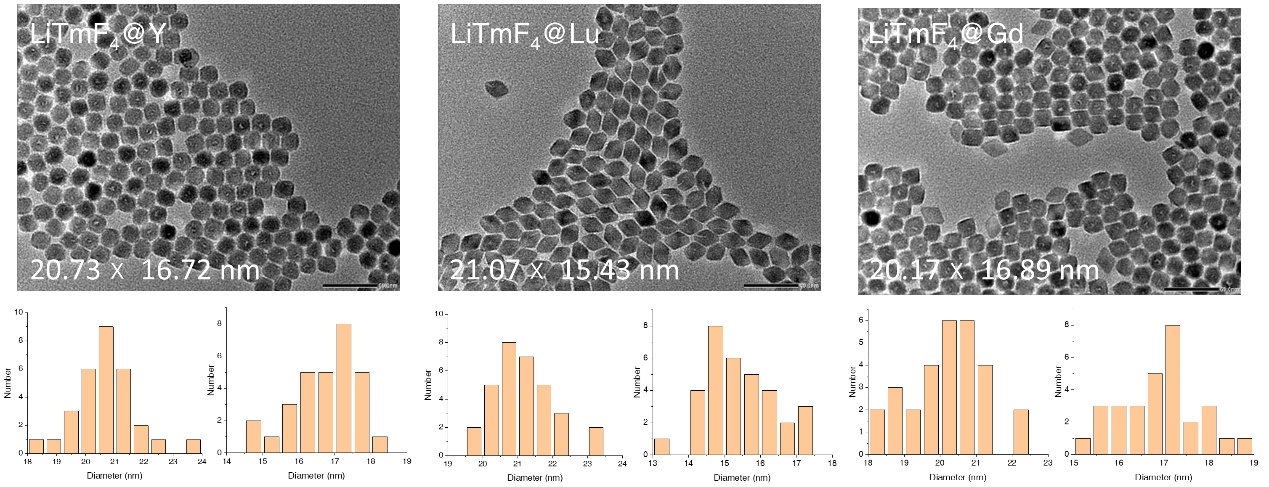


**Supplementary Fig. 13.** TEM images of LiTmF_4_@Y, LiTmF_4_@Lu, and LiTmF_4_@Gd core-shell NPs prepared with the same LiTmF_4_ core NPs. Scale bars are 50 nm.


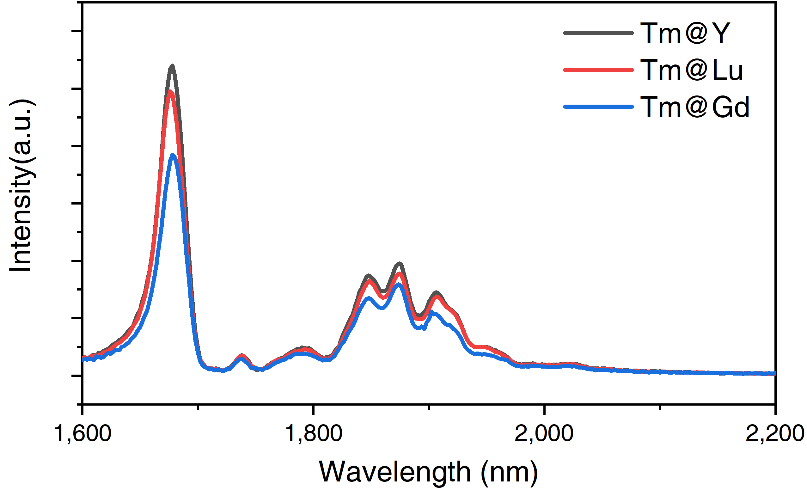


**Supplementary Fig. 14.** Emission spectra of LiTmF_4_@LiYF_4_, LiTmF_4_@LiLuF_4_, and LiTmF_4_@LiGdF_4_ core-shell NPs in cyclohexane under 800 nm light irradiation.

Comparing the luminescence intensities showed that the LiYF_4_ and LiLuF_4_ shells presented similar luminescence intensities (that for Y was slightly stronger), while the LiGdF_4_ shell resulted in the weakest DSL. This was mainly because of the close proximities of the Y and Lu shells to the LiTmF_4_ core, which resulted in minor lattice mismatches and fewer interface defects but stronger emission^4^.


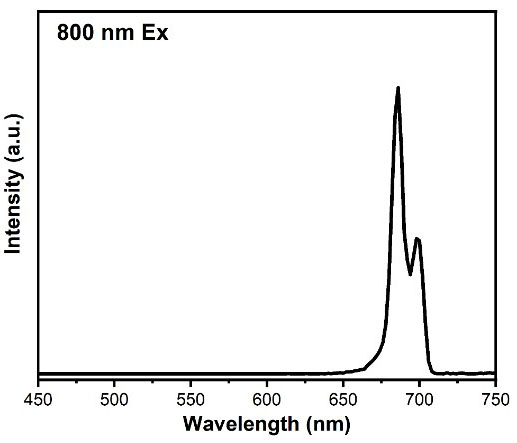


**Supplementary Fig. 15.** Upconversion luminescence of LiTmF_4_@LiYF_4_ NPs in cyclohexane under 800 nm irradiation.

The Tm-NPs showed predominantly monochromatic red emission at 696 nm, similar to that reported for single Er-NPs (NaErF_4_@NaYF_4_)^5^. Nevertheless, this can only be measured with high excitation power due to its low luminescence efficiency. Based on the mechanism for downshifted luminescence of Tm^3+^, we deduced that increasing the doping concentration increased the CR effect, inhibited upconversion and facilitated the ^3^F_4_→^3^H_6_ transition.


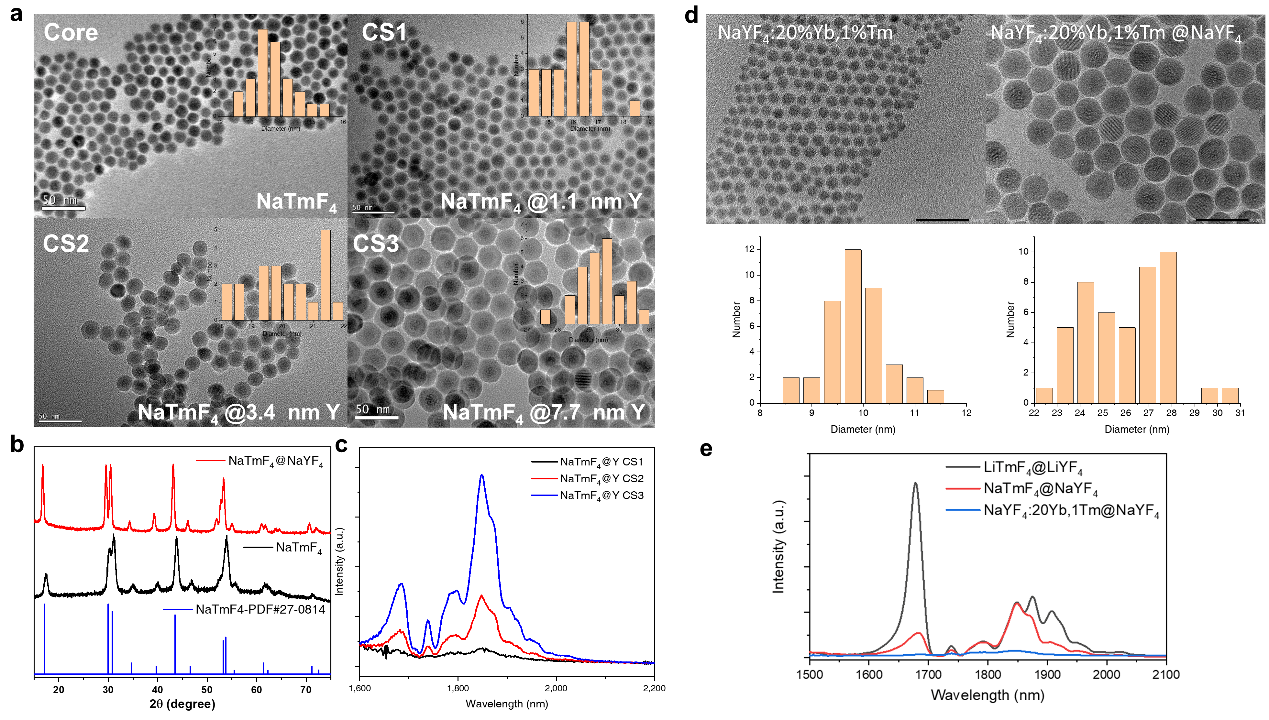


**Supplementary Fig. 16.** **a**, TEM images of NaTmF_4_ core and NaTmF_4_@NaYF_4_ core-shell NPs with the size of 13.69 ± 2.1 nm, 18.13 ± 1.7 nm, 26.9 ± 1.1 nm. **b,** XRD patterns of the NaTmF_4_ and NaTmF_4_@NaYF_4_ NPs and the standard patterns of β-NaTmF_4_. **c,** Emission spectra of NaTmF_4_@NaYF_4_ NPs with different shell thicknesses in cyclohexane under 800 nm light irradiation. **d**, TEM images of NaYF_4_:20%Yb,1%Tm@ NaYF_4_ core and NaYF_4_:20%Yb,1%Tm@NaYF_4_ core-shell NPs with the size of 26.02 ± 2.1 nm. **e**, Comparison of emission profiles of Tm-based NPs with a similar size using the same excitation power density (980 nm light for Yb/Tm NPs, 800 nm light for Na- and Li-based Tm-NPs).


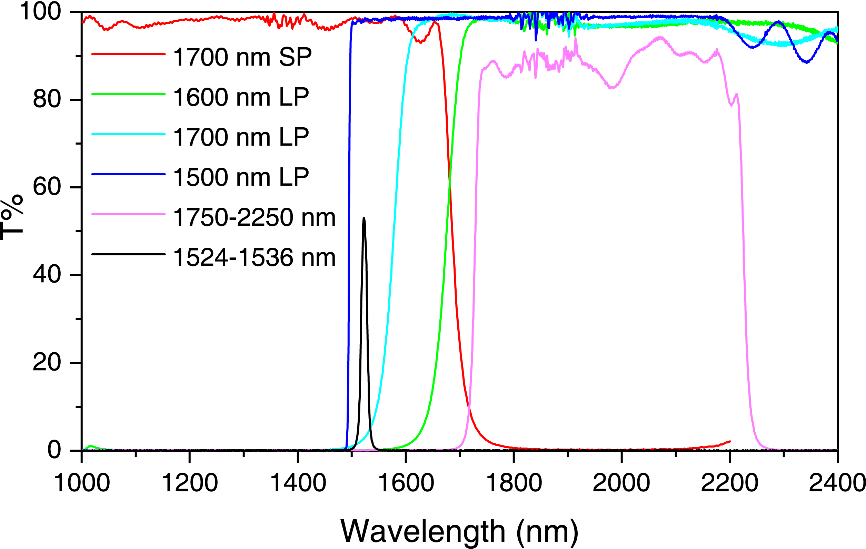


**Supplementary Fig. 17**. Transmittance window of various used filters, including shortpass filter of 1,700 nm SP (custom made) with a cut-off wavelength of 1,700 nm, longpass filters of 1,500 nm LP (FELH 1,500, Thorlabs), 1,600 nm LP (custom made), 1,700 nm LP (custom made) with a cut-on wavelength of 1,500, 1,600 and 1,700 nm, respectively, bandpass filters of FB2000-500 (Thorlabs) and FB1,530-12 (Thorlabs) with transmittance window at 1,750-2,250 nm and 1,524-1,536 nm, respectively.


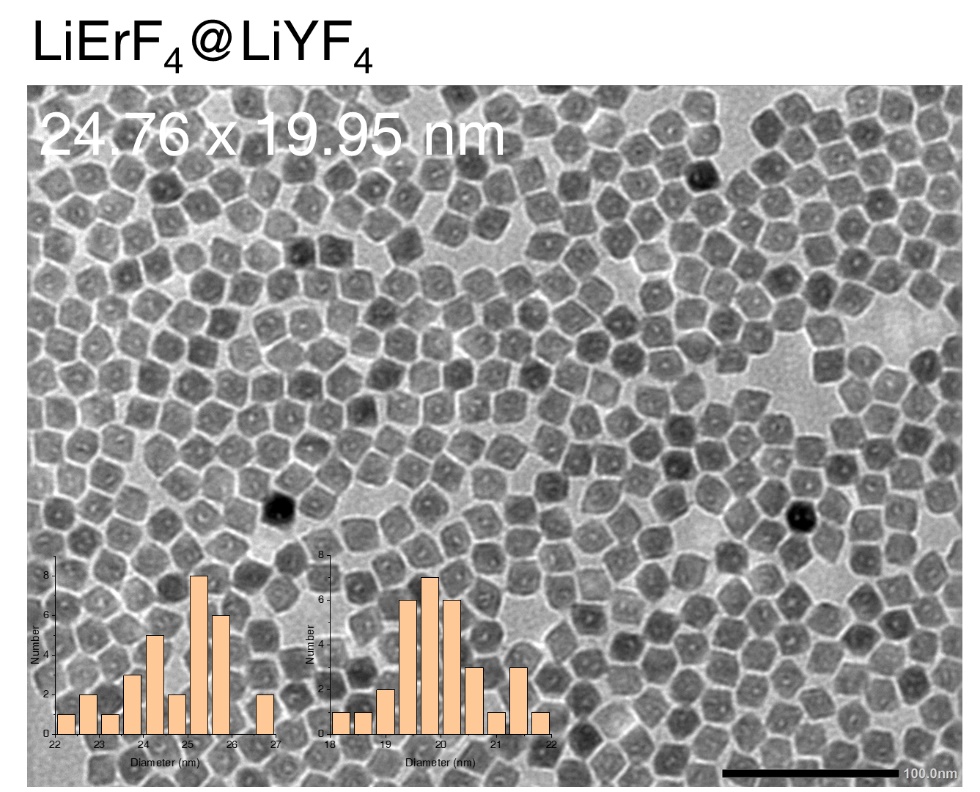


**Supplementary Fig. 18**. TEM image of LiErF_4_ @LiYF_4_ core-shell NPs. Scale bar =100 nm.


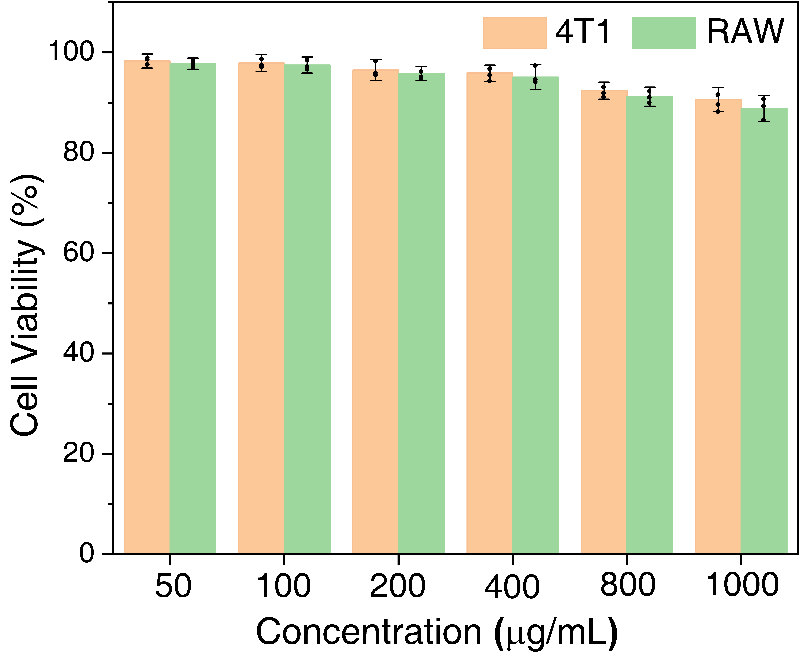


**Supplementary Fig. 19.** Cell viabilities of 4T1 cells and Raw cells with different concentrations of Tm-based probe treatment, data are presented as mean ± s.d. (n=3).


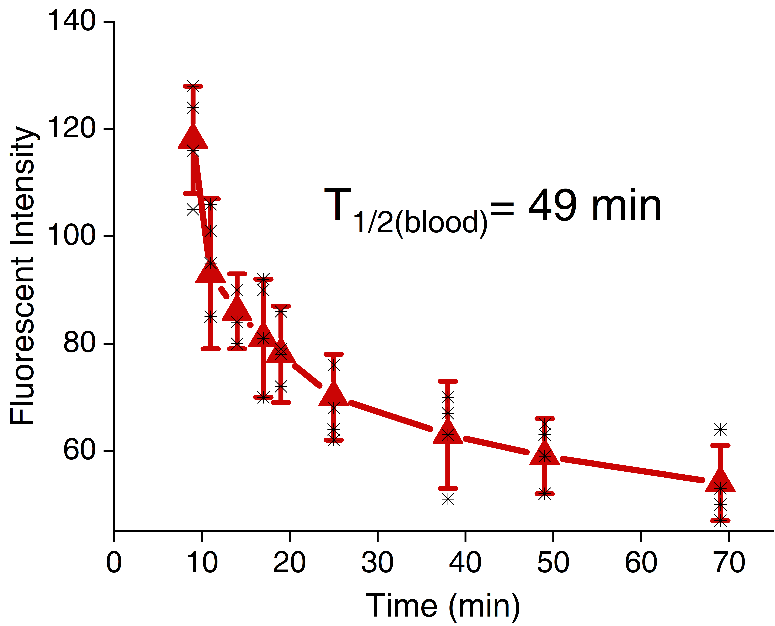


**Supplementary Fig. 20.** Bloold circulation of Tm-based nanoprobe administrated mice as a function of time, data are presented as mean ± s.d. (n=4).


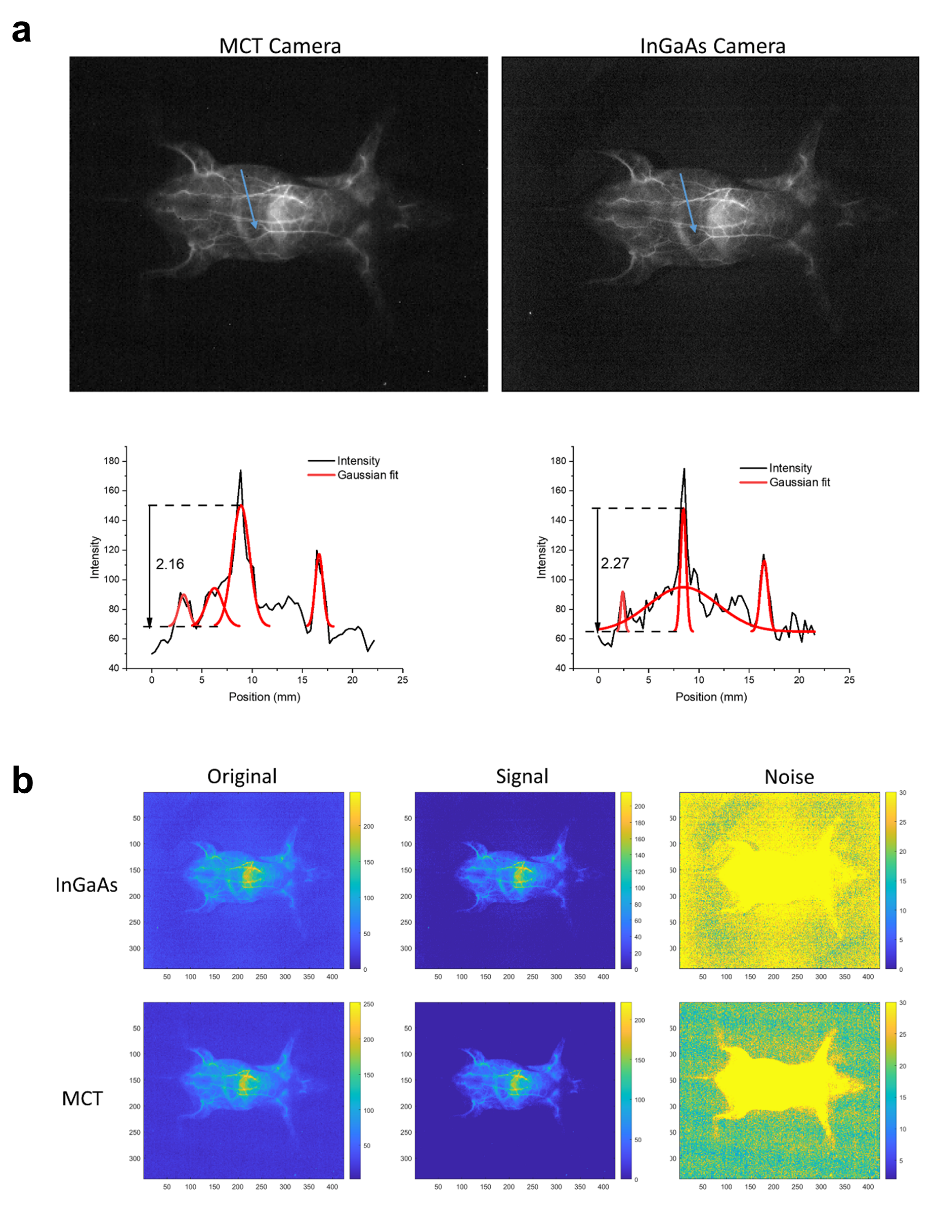


**Supplementary Fig. 21.** **a.** Comparison of NIR-IIb luminescence imaging of blood vessels in the same mouse, treated with Tm(02Er)-NPs@PEG under 800 nm excitation (200 mW/cm^2^) with 1,600 nm LP and 1,700 nm SP filters. Corresponding cross-sectional luminescence intensity profiles along blue lines. Gaussian fits are shown in the red line. **b.** The blood vessel imaging with InGaAs and MCT camera of the original image, target signal and noise (analyzed with Matable R2021a).


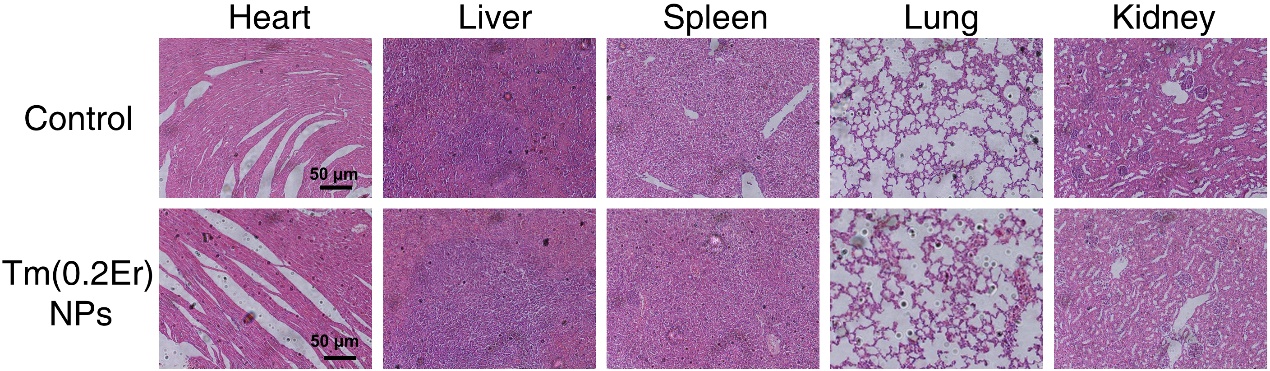


**Supplementary Fig. 22.** H&E staining of major organs after treatment with PBS and Tm(0.2Er)-NPs@PEG nanoprobe.


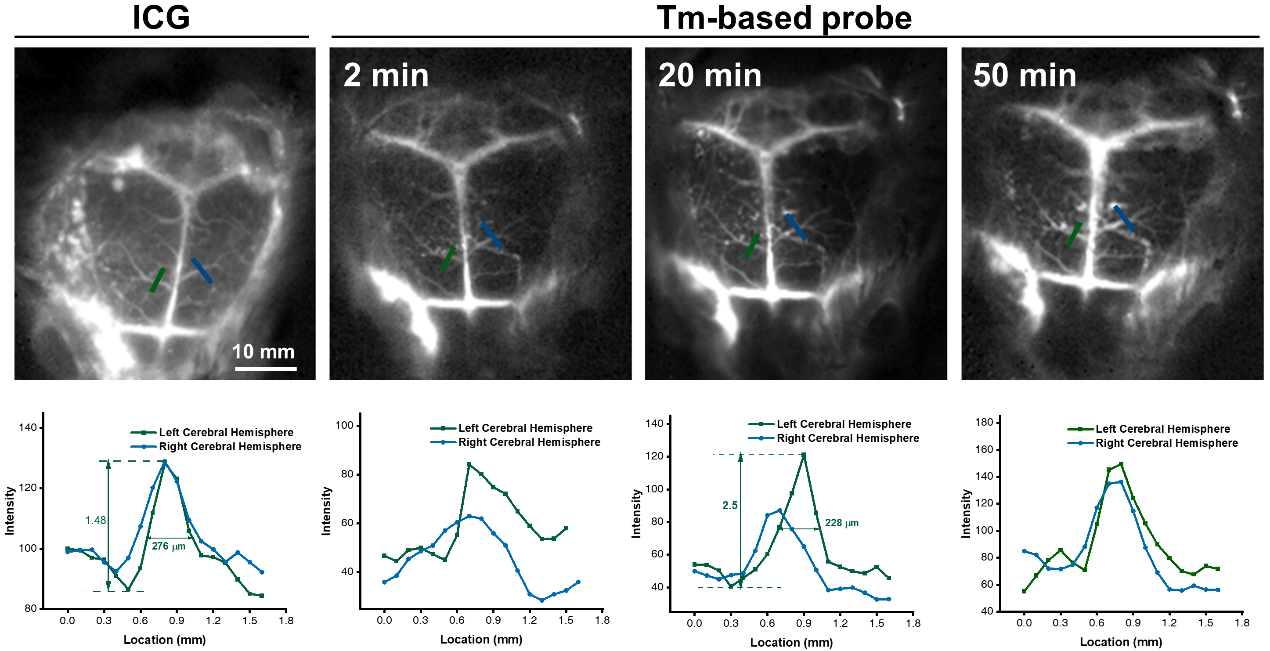


**Supplementary Fig. 23.** NIR-II fluorescence imaging of mouse cerebral vasculature obtained by an MCT camera under 800 nm excitation. Faster metabolized ICG dye with a short half-life (3-5 min) as the control for NIR-IIa imaging (1,300 nm LP filter) and Tm-based NPs for NIR-IIc imaging (1,700 nm LP filter). Scale bars are 10 mm.

Pre-MCAO surgery, a short blood half-life (3-5 min) of FDA-approved dye, ICG^6^, was intravenously injected into the mouse for imaging as control. With ICG, 276 µm vessels could be detected with SBR=1.48 by NIR-IIa (1,300 nm LP filter) imaging. After 2 h metabolism, the Tm-based probes were injected into the same mouse with the MCAO model and then exposed to 800 nm light again for NIR-IIc imaging. NIR-IIc imaging could more clearly distinguish blood vessels (FWHM= 228 µm) with SBR= 2.5. Notably, it could be found that several blood vessels in the right cerebral hemisphere were dimmed compared to the situation before the MCAO model was established or the left cerebral hemisphere. Afterwards, the structure and shape of blood vessels became invisible at 20 min time points, implying that thrombosis blocked the passage of blood. However, after 60 min postinjection, the right cerebral hemisphere becomes brighter due to the self-protective mechanism of collateral circulation. The corresponding statistical analysis of vascular luminescence intensities further confirmed the successful establishment of the thrombus model (weakened) and the formation of collateral circulation (restored).


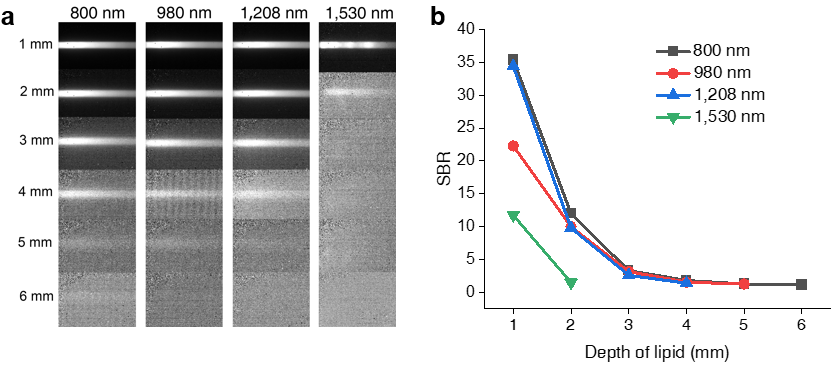


**Supplementary Fig. 24. a,** Luminescence imaging of a capillary tube filled with Tm(30Er) NPs aqueous solution, immersed at different depths (1 mm, 2 mm, 3 mm, 4 mm, 5 mm and 6 mm) in 1% intralipid solution and recorded at NIR-IIb and NIR-IIc emissions upon 800 nm, 980 nm, 1,208 nm and 1,530 nm excitation, respectively. **b**, the corresponding SBR luminescence intensity profiles of **a**.

**Supplementary References**

1. Benson RC, Kues HA. Fluorescence properties of indocyanine green as related to angiography. *Physics in Medicine and Biology* **23**, 159-163 (1978).

2. Chen G*, et al.* (alpha-NaYbF4:Tm(3+))/CaF2 core/shell nanoparticles with efficient near-infrared to near-infrared upconversion for high-contrast deep tissue bioimaging. *ACS Nano* **6**, 8280-8287 (2012).

3. Wang X*, et al.* Efficient Erbium-Sensitized Core/Shell Nanocrystals for Short Wave Infrared Bioimaging. *Adv Opt Mater* **6**, 1800690 (2018).

4. Zhang Y, Zhu X, Zhang Y. Exploring Heterostructured Upconversion Nanoparticles: From Rational Engineering to Diverse Applications. *ACS Nano* **15**, 3709-3735 (2021).

5. Zuo J*, et al.* Employing shells to eliminate concentration quenching in photonic upconversion nanostructure. *Nanoscale* **9**, 7941-7946 (2017).

6. Chen H*, et al.* Smart Self-Assembly Amphiphilic Cyclopeptide-Dye for Near-Infrared Window-II Imaging. *Adv Mater*, e2006902 (2021).
