# Supplementary material for: Bright Tm3+-based downshifting luminescence nanoprobe operating around 1800 nm for NIR-IIb and c bioimaging
Source: Nat Commun. 2023 Feb 25;14:1079. doi: 10.1038/s41467-023-36813-5 (PMC9968279; doi:10.1038/s41467-023-36813-5)
Supplement: Supplementary file 3 — Reporting Summary [file 41467_2023_36813_MOESM3_ESM.pdf]

nature portfolio

Corresponding author(s): Yulei Chang
Last updated by author(s): Dec 23, 2022

Reporting Summary

Nature Portfolio wishes to improve the reproducibility of the work that we publish. This form provides structure for consistency and transparency in reporting. For further information on Nature Portfolio policies, see our Editorial Policies and the Editorial Policy Checklist.

Statistics

For all statistical analyses, confirm that the following items are present in the figure legend, table legend, main text, or Methods section.

n/a	Confirmed	
☐	☒	The exact sample size (*n*) for each experimental group/condition, given as a discrete number and unit of measurement
☐	☒	A statement on whether measurements were taken from distinct samples or whether the same sample was measured repeatedly
☒	☐	The statistical test(s) used AND whether they are one- or two-sided<br>*Only common tests should be described solely by name; describe more complex techniques in the Methods section.*
☒	☐	A description of all covariates tested
☒	☐	A description of any assumptions or corrections, such as tests of normality and adjustment for multiple comparisons
☐	☒	A full description of the statistical parameters including central tendency (e.g. means) or other basic estimates (e.g. regression coefficient) AND variation (e.g. standard deviation) or associated estimates of uncertainty (e.g. confidence intervals)
☒	☐	For null hypothesis testing, the test statistic (e.g. *F*, *t*, *r*) with confidence intervals, effect sizes, degrees of freedom and *P* value noted<br>*Give P values as exact values whenever suitable.*
☒	☐	For Bayesian analysis, information on the choice of priors and Markov chain Monte Carlo settings
☒	☐	For hierarchical and complex designs, identification of the appropriate level for tests and full reporting of outcomes
☒	☐	Estimates of effect sizes (e.g. Cohen's *d*, Pearson's *r*), indicating how they were calculated

Our web collection on statistics for biologists contains articles on many of the points above.

Software and code

Policy information about availability of computer code

Data collection	F980 (Edinburgh Instruments)1.4.1.0; PHyspecV2 (Photon Etc);Zetasizer Software 7.13 (Malvern); Ocean QY 2.02
Data analysis	The FWHM was measured in OriginPro 2021.<br>Fluorescence intensity data was processed in ImageJ 1.51.<br>Spectral data are processed with OriginPRO 2021.<br>SBR are processed with Matlab 2021.

For manuscripts utilizing custom algorithms or software that are central to the research but not yet described in published literature, software must be made available to editors and reviewers. We strongly encourage code deposition in a community repository (e.g. GitHub). See the Nature Portfolio guidelines for submitting code & software for further information.

Data

Policy information about availability of data

All manuscripts must include a data availability statement. This statement should provide the following information, where applicable:

- Accession codes, unique identifiers, or web links for publicly available datasets
- A description of any restrictions on data availability
- For clinical datasets or third party data, please ensure that the statement adheres to our policy

The experimental data supporting the findings of this study are available within the article, Supplementary Information, and Source Data. All relative data are

available from the corresponding authors upon request. Source data are provided with this paper.

Human research participants

Policy information about studies involving human research participants and Sex and Gender in Research.

Reporting on sex and gender	N/A
Population characteristics	N/A
Recruitment	N/A
Ethics oversight	N/A

Note that full information on the approval of the study protocol must also be provided in the manuscript.

Field-specific reporting

Please select the one below that is the best fit for your research. If you are not sure, read the appropriate sections before making your selection.

- [x] Life sciences
- [ ] Behavioural & social sciences
- [ ] Ecological, evolutionary & environmental sciences

For a reference copy of the document with all sections, see nature.com/documents/nr-reporting-summary-flat.pdf

Life sciences study design

All studies must disclose on these points even when the disclosure is negative.

Sample size	No sample size calculations were performed. Animal sample size was determined by the number of biological replicates necessary for ensuring statistical significance. Three 20 g Balb/c mice were randomly selected for all animal imaging experiments. The cytotoxicity test was conducted with 3 wells in each group.
Data exclusions	No data were excluded from the analyses.
Replication	All experiments were carried out at least 3 times successfully.
Randomization	In order to ensure the repeatability of the experiment, animal experiments and other experiments mentioned are conducted in random groups.
Blinding	The investigators were blinded to group allocation during data collection and/or analysis

Reporting for specific materials, systems and methods

We require information from authors about some types of materials, experimental systems and methods used in many studies. Here, indicate whether each material, system or method listed is relevant to your study. If you are not sure if a list item applies to your research, read the appropriate section before selecting a response.

Materials & experimental systems

n/a	Involved in the study	
x		Antibodies
	x	Eukaryotic cell lines
x		Palaeontology and archaeology
	x	Animals and other organisms
x		Clinical data
x		Dual use research of concern

Methods

n/a	Involved in the study	
x		ChIP-seq
x		Flow cytometry
x		MRI-based neuroimaging

Eukaryotic cell lines

Policy information about cell lines and Sex and Gender in Research

Cell line source(s)	RAW264. 7 cells (Leukemia cells in mouse macrophage) and 4T1 cells (Murine breast carcinoma cells) were obtained from the Institute of Basic Medicine, Chinese Academy of Medical Science.
Authentication	These cells were authenticated by cell vitality test, isozyme detection, and mycoplasma detection.
Mycoplasma contamination	The cell lines were detected for mycoplasma contaminaiton and no mycoplasma was found.
Commonly misidentified lines (See ICLAC register)	None of these cell lines were used.

Animals and other research organisms

Policy information about studies involving animals; ARRIVE guidelines recommended for reporting animal research, and Sex and Gender in Research

Laboratory animals	BALB/c mice (6-8 weeks old) were used in this paper. All mice were housed in a specific pathogen-free environment at 26+/- 1°C and 50 +/- 5% humidity, with a 12 h light-dark cycle.
Wild animals	The study does not involve wild animals.
Reporting on sex	The sex of mice was not involved in the study.
Field-collected samples	No field-collected samples were used
Ethics oversight	The Animal Use and Care Committee at Northeastern University approved all of the experimental procedures, which followed the National Institutes of Health Guidelines.

Note that full information on the approval of the study protocol must also be provided in the manuscript.
